# Puerarin Prevents Bisphenol S Induced Lipid Accumulation by Reducing Liver Lipid Synthesis and Promoting Lipid Metabolism in C57BL/6J Mice

**DOI:** 10.3390/toxics11090736

**Published:** 2023-08-26

**Authors:** Zi-Yao Wu, Li Luo, Ya-Qi Kan, Mei-Lin Qin, Hai-Ting Li, Qing-Zhi He, Huai-Cai Zeng

**Affiliations:** 1 Guangxi Key Laboratory of Environmental Exposomics and Entire Lifecycle Health, School of Public Health, Guilin Medical University, Guilin 541199, China; ziyaowu@163.com (Z.-Y.W.); ll58654118@163.com (L.L.); kyq8426@163.com (Y.-Q.K.); qmeilin2022@163.com (M.-L.Q.); serendipity0803@163.com (H.-T.L.); 2 Guangxi Health Commission Key Laboratory of Entire Lifecycle Health and Care, School of Public Health, Guilin Medical University, Guilin 541199, China; 3 College of Intelligent Medicine and Biotechnology, Guilin Medical University, Guilin 541199, China

**Keywords:** bisphenol S, puerarin, lipid accumulation, lipid metabolism

## Abstract

Bisphenol S (BPS) is an environmental pollutant that can accumulate in the human body and cause harm. Puerarin (PUE) is a flavonoid with anti-inflammatory and antioxidant effects. In this study, we used 50 mg/kg/d BPS as a poison and PUE as an intervention for model mice for 42 d. BPS exposure significantly increased the levels of the impairment of the mice’s liver function, T-CHO, TG, LDL-C, ALT, and AST in the BPS group were significantly increased (*p* < 0.05). Additionally, BPS exposure caused inflammatory cell infiltration in the mice liver tissue and enhanced oxidative stress response, the level of MDA was significantly increased (*p* < 0.05). The expression of CD36 and pparγ was stimulated after BPS exposure. Moreover, the expression of cpt1a and cpt1b, which promote fatty acid oxidation, was downregulated. After PUE intervention, the levels of genes and proteins involved in lipid synthesis (PPARγ, SREBP1C, and FASN) and metabolism (Cpt1a, Cpt1b, and PPARα) in mice returned to those of the control group, or much higher than those in the BPS group. Therefore, we hypothesized that BPS causes lipid accumulation in the liver by promoting lipid synthesis and reducing lipid metabolism, whereas PUE reduces lipid synthesis and promotes lipid metabolism. Conclusively, our results imply that long-term exposure to BPS in mice affects liver lipid metabolism and that PUE intervention could maintain the liver function of mice at normal metabolic levels.

## 1. Introduction

After the ban on bisphenol A (BPA), alternative compounds have been widely used. Bisphenol S (BPS) is freely soluble in aliphatic hydrocarbons [1], and was first manufactured as a dye in 1869 and used for cash register receipts in 2006 [2]. BPS is an alternative to BPA that is used in various products. The median value of BPS metabolite BPS-G detected in the urine of the general population in Guangzhou, China is 0.38 ng/mL [3], but in other studies, the median concentration of BPS in the urine of special occupational groups such as cashiers exposed to thermal paper is 2.53 μg/L [4], which is much higher than that of the general population. Basic studies have shown that BPS can affect the secretion of the inflammatory cytokines interleukin (IL)-6 and IL-8 in HTR-8/Svneo cells more than BPA [5], and the reproducibility of BPS in human urine is higher than that of BPA [6], indicating that BPS is likely to increase in concentration in various media over time. It can be seen that BPS has no lesser impact on health than BPA.

An increasing number of studies have demonstrated that phenol exposure is associated with obesity [7,8]. In a comparison of different statistical models, BPA and BPS have been identified as the most important factors associated with obesity [9], as they can cause obesity by interfering with lipid metabolism and promoting lipid accumulation and fat production [7,10,11]. At the same time, obesity is often accompanied by liver metabolic disorders and local or systemic inflammatory reactions [12,13]. The liver is the central organ involved in fatty acid (FA) metabolism. It can oxidize lipids and pack excess lipids into adipose tissue [14]. Fatty acid synthase (FASN) is a central regulator of lipid metabolism and a downstream lipase of sterol regulatory element-binding proteins (SREBPs). Multiple genes activated by SREBPs are involved in the uptake and synthesis of total cholesterol (T-CHO), FAs, and TG [15]. The cluster of differentiation 36 (CD36) scavenger receptor is a key regulator of de novo lipogenesis (DNL) in liver cells and promotes tissue FA uptake. The downregulation or inhibition of CD36 reduced ochratoxin A-induced lipid droplet deposition and TG accumulation [16]. Studies have found that the overexpression of CD36 stimulates insulin-mediated DNL and in vitro fat droplet formation [17]. Peroxidase proliferation activation receptors (PPARs) are ligand-induced transcription factors that play a key role in lipid metabolism. PPARα participates in the regulation of inflammation, nutritional metabolism, and the steady state. It is the main activator of FA oxidation in the liver, heart, and brown adipose tissue [18]. Research has shown that the long-lived enzyme Sirtuin 6 (SIRT6) promotes liver beta-oxidation by activating PPARα and mediates PPARα to inhibit SREBP-dependent cholesterol and TG synthesis [19]. PPARγ is highly expressed in adipose tissue and regulates fat production, lipid metabolism, and insulin sensitivity. Its upward adjustment promotes higher levels of fat in the liver and contributes to higher body fat accumulation in cavefish stocks [20]. However, it has been found that the upregulation of PPARγ can reduce serum aspartate aminotransferase (AST), alanine aminotransferase (ALT), total bilirubin, and pathological liver damage in mice and improve their survival rate [21]. Thus, PPARγ plays different roles in different species or under different conditions in the same species. Carnitine palmitoyltransferase a (Cpt1a) and carnitine palmitoyltransferase b (Cpt1b) are two subtypes of carnitine palmitoyltransferase-1 (CPT-1). These are target genes of PPARα and they participate in fatty acid oxidation [22]. Puerarin (PUE), isolated from Pueraria lobata (Wild), is the main bioactive component of the Pueraria lobata root and has a wide range of pharmacological effects [23], such as neuroprotection, anti-oxidation, and anti-inflammation [24]. Studies have shown that PUE can partially improve myocardial ischemia by regulating FA accumulation in rats [25]. Liu et al. showed that PUE could reduce lipid accumulation, T-CHO, and TG levels and alleviate alcohol-induced hepatic steatosis in zebrafish larvae by regulating alcohol and lipid metabolism [26]. PUE or PUE extract can improve inflammation and lipid peroxidation in mice fed alcohol and high-fat diets and improve non-alcoholic fatty liver disease induced by a high-fat diet [27,28]. However, the mechanisms underlying the effects of PUE on BPS-induced lipid metabolism in the liver remain unclear. Therefore, to explore whether the liver lipid metabolism of long-term exposure to bisphenol mice has changed, this experiment used BPS (50 mg/kg/day) and PUE (150 mg/kg/day) to gavage mice for 42 d; Western blotting and real-time PCR were used to studying the protein or messenger ribonucleic acid (mRNA) expression changes of PPARγ, CD36, SREBP1C, and FASN which involved in lipid and sterol synthesis, and PPARα, Cpt1a, and Cpt1b which involved in FA oxidation, and the liver injury of mice was evaluated by histological staining (H&E and oil red). At the same time, detecting mouse serum liver function-related indicators by kits, such as ALT and AST, and oxidative stress indicators, such as malondialdehyde (MDA) and superoxide dismutase (SOD). We explored the effect of BPS and PUE on liver lipid metabolism in C57BL/6J mice, this paper lays a foundation for better elucidating the mechanism of BPS-induced lipid metabolism disorders in mice and studying the role of PUE in lipid metabolism disorder intervention.

## 2. Materials and Methods

### 2.1. Animal Experiments

**Figure 1:**
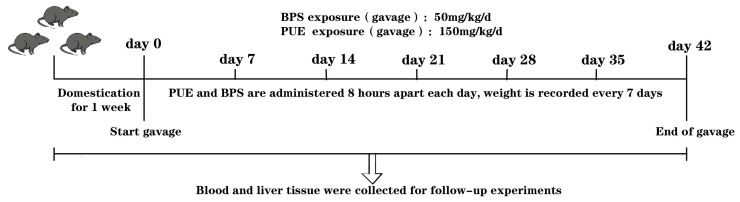
The technology roadmap.

In total, 30 healthy adult specific-pathogen-free (SPF)-level C57BL/6J male eight-week-old mice with a weight of 18–22 g were randomly divided into three groups (*n* = 10): control, BPS (50 mg/kg/d), and PUE + BPS (150 mg/kg/d + 50 mg/kg/d). The mice were raised in the Experimental Animal Center of Guilin Medical College (12 h dark/light circulation; relative humidity: 40–70%; room temperature: 23 ± 2 °C; free access to food and water). PUE was dissolved in double-steamed water and BPS was dissolved in sesame oil. The daily BPS group was administered 50 mg/kg BPS for gastric irrigation, the PUE group was first administered 150 mg/kg PUE for gastric irrigation, and 50 mg/kg BPS was administered after 8 h. The control group was administered an equal amount of sesame oil and dissected after 42 consecutive days of gastric irrigation. The technology roadmap is shown in Figure 1. All animal experiments were approved by the Animal Ethics Committee of Guilin Medical College (GLMC-IACUC-2022030).

### 2.2. Sample Collection In Vivo

#### 2.2.1. Blood Sampling

The mice were intraperitoneally injected with 40 mg/kg pentobarbital sodium. After anesthesia, the abdomen was cut open, and the abdominal aorta was isolated. The disposable 1 mL syringe needle is pierced in parallel into the abdominal aorta. The needle is fixed with one hand and the syringe is slowly pumped with the other hand to draw the required amount of blood (0.6–0.8 mL). After standing for 30 min, the blood was centrifuged at 3000 rpm for 10 min in a 1.5 mL Eppendorf (EP) tube and the upper serum was pipetted into a new EP tube and stored at −80 °C refrigerator.

#### 2.2.2. Organ Sampling

After the liver was removed, the blood on its surface was dried using filter paper, weighed, and recorded. The mouse liver was separated and placed in different EP tubes and transported to −80 °C refrigerator by liquid nitrogen. In addition, the liver caudate lobe tissues of 6 mice in each group were randomly selected and used, respectively, for Oil Red O staining (*n* = 3) and hematoxylin and eosin (H&E) staining (*n* = 3).

### 2.3. Histological Stains

#### 2.3.1. Oil Red O Stain

The liver was placed in an embedding mold, covered with SAKURA Tissue-Tek^®^ O.C.T. Compound (4583, SAKURA, Torrance CA, USA), wrapped in tin paper, and placed on the surface of liquid nitrogen for cooling and coagulation. After complete coagulation, slices that were 8 μm each were made using a frozen section machine (CM1950, Leica, Nussloch, Germany). According to the instructions of the modified Oil Red O kit (G1262, Solarbio, Beijing, China), the slides with adsorbed liver tissue sections were placed in 60% isopropanol for 2 min, taken to ice water (ice water mixture) for slight cleaning, put into the Oil Red O staining solution for 10 min (away from light), put into 60% isopropanol for 5 s, and washed with ice water; finally, hematoxylin staining was used to stain the nucleus for 5 min, and the edge of the tissue was dried after ice water washing. Glycerin gelatin was used to seal the film, and photographs were obtained under an inverted microscope.

#### 2.3.2. H&E Stain

The freshly collected liver samples were rinsed, dried, fixed in 4% paraformaldehyde, and then embedded in paraffin. Treated liver sections (6–8 μm) were dewaxed by xylene (2 times, each for 10 min), absolute ethanol (2 times, each for 5 min), ethanol of different concentrations (95%, 90%, 80%, 70%), and water separately. Then, placed it in hematoxylin for 5 min to stain the nucleus, rinsed with running water, and stained with eosin for 1 min to stain the cytoplasm. The slices were dehydrated in anhydrous ethanol 3 times (each for 5 min), Finally, xylene was used for permeabilization 3 times (each for 10 min) and sealed with neutral gum after drying. Representative sections were imaged using an inverted microscope.

### 2.4. Measurements of Biochemical Parameters and Oxidative Stress Parameters

Serum ALT (C009-2-1), AST (C010-2-1), TG (A110-1-1), T-CHO (A111-1-1), high-density lipoprotein cholesterol (HDL-C, A112-1-1), and low-density lipoprotein cholesterol (LDL-C, A113-1-1) levels were measured according to the manufacturer’s instructions. Liver homogenates were detected according to the manufacturer’s instructions, the WST-1 method was used to detect SOD levels (A001-3), catalase (CAT, A007-1-1) was tested by the ammonium molybdate method, reduced glutathione (GSH, A006-2-1) was tested by DTNB method, and MDA (BC0025) was tested by TBA method. All the kits used a full-wavelength scanning multifunction microplate reader (Thermo scientific Varioskan LUX, Waltham, MA, USA) to quantify. All the kits were purchased from Nanjing Institute of Bioengineering, China.

### 2.5. Quantitative Reverse Transcription Polymerase Chain Reaction (RT-PCR)

Total RNA from the mouse liver tissue was extracted using the Trizol (15596026, ambion, Carlsbad CA, USA) method. After reverse transcription with the kit (MR05101, Monad, SuZhou, China), the two-step method was used to detect the mRNA levels of PPARα, PPARγ, CD36, SREBP1C, Cpt1a, Cpt1b, FASN, tumor necrosis factor-α (TNF-α), and IL-1β by fluorescence quantitative instrument (QuantStudio6Flex). Then, it underwent 95 °C pre-denaturation for 30 s, 95 °C denaturation for 10 s, 60 °C annealing and extension for 30 s, followed by 40 cycles of this. Relative expression levels of each target gene were calculated using the 2^−ΔΔCt^ method [29]. The primer sequences are listed in Table 1.

### 2.6. Western Blotting

The total protein in the mouse liver tissue was extracted, and the protein concentration was determined using a Bicinchoninic acid (BCA) kit (P0010S, Beyotime, Shanghai, China). After the total protein concentration was adjusted, the protein-loading buffer was added and boiled in a 100 °C water bath to obtain the target sample. Gel electrophoresis was performed using 8% concentrated gel and separation gel (G2042-4, servicebio, Wuhan, China). Proteins were transferred to a polyvinylidene fluoride (PVDF, IPVH00010, servicebio, Wuhan, China) membrane and blocked with a rapid blocking solution at room temperature for 1 h. The PVDF membrane was incubated with PPARγ (16643-1-AP, 1:7500, Proteintech Group, Inc, Wuhan, China), SREBP1C (66875-1IG, 1:2500, Proteintech Group, Inc., Wuhan, China), Cpt1b (22170-1-AP, 1:5000, Proteintech Group, Inc., Wuhan, China), and FASN (GB11546, 1:1000, servicebio, Wuhan, China) protein primary antibodies and incubated in a refrigerator at 4 °C overnight, incubated with goat anti-rabbit (#S0001, 1:7500, servicebio, Wuhan, China) or goat anti-mouse (#S0001, 1:5000, servicebio, Wuhan, China) secondary antibody on a shaker at room temperature for 1 h, washed with 0.1% Tween^®^ 20 (T8220, Solarbio, Beijing, China) detergent (TBST) solution, detected the expression of the target protein by enhanced chemiluminescence reagent and chemiluminescence imaging system, and analyzed the gray value by Image J 1.53a software; 3 repeated tests were completed.

### 2.7. Statistical Analysis

Statistical analyses were performed using International Business Machines (IBM) SPSS Statistics 21 software. All quantitative data were presented as (Mean ± Standard Error of the Mean [SEM]). Statistical significance was identified by analysis of variance (ANOVA) followed by the least significant difference (LSD) test. Use the Shapiro–Wilk test to detect data normality and Levene’s test to detect variance homogeneity. When the data did not meet the normality and homogeneity of variance, Kruskal–Wallis analysis was used to compare the differences between the groups. GraphPad Prism 8.0.2 and Adobe Illustrator CS6 were used to draw illustrations. “*” represented a statistically significant difference between the BPS group and the control group (*p* < 0.05), “#” represented a statistically significant difference between the BPS group and the PUE + BPS group (*p* < 0.05).

## 3. Results

### 3.1. Body Weight and Organ Coefficient

**Figure 2:**
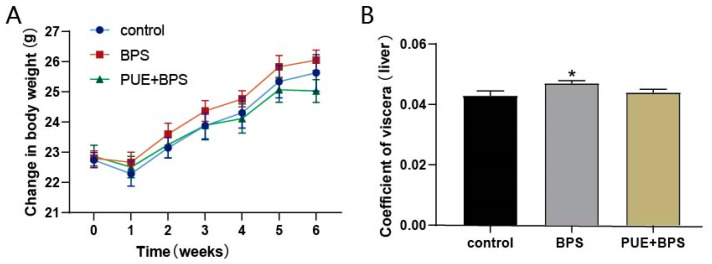
(**A**) Weight changes of mice in each group for 6 weeks. (**B**) Liver coefficient (liver weight/body weight). *n* = 10. “*” represents the comparison between the BPS group and the control group, *p* < 0.05.

In the first week of gavage, the weight of the mice in each group decreased slightly and gradually increased after a week of adaptation. Compared with the control group, the weight gain of mice in the BPS group was more obvious, but there was no significant difference in the weight of mice in each group, and the weight change of mice in the PUE + BPS group was roughly the same as that in the control group, and even gradually lower than that in the control group after the fifth week (Figure 2A). The liver coefficient of mice in the BPS group was higher than that of mice in the control and PUE + BPS groups (Figure 2B). It may have increased weight owing to liver fat accumulation, and PUE can relieve the weight gain induced by BPS.

### 3.2. Changes of Serum Lipid and Liver Function Indexes in Each Group

**Figure 3:**
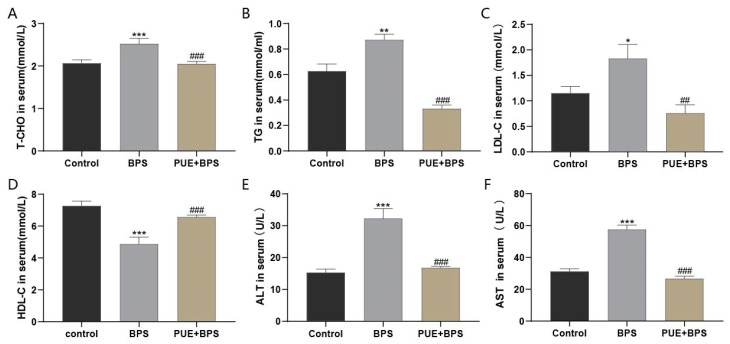
Comparison of blood lipid levels in three groups of different mice. (**A**) Total cholesterol; (**B**) triglycerides; (**C**) low-density lipoprotein cholesterol; (**D**) high-density lipoprotein-cholesterol; (**E**) alanine aminotransferase; (**F**) aspartate aminotransferase; “*”, “**” and “***” represents the comparison between the BPS group and the control group, *p* < 0.05, *p* < 0.01 and *p* < 0.001, separately; “##“ and “###” represents the comparison between the BPS group and the PUE + BPS group, *p* < 0.01, *p* < 0.001, separately. *n* = 6.

To explore whether BPS exposure caused liver damage, we measured the levels of serum biochemical indicators in mice. Compared to the control group, the levels of T-CHO, TG, LDL-C, ALT, and AST in the BPS group were significantly increased (Figure 3A–C,E,F), and the level of HDL-C was significantly decreased (Figure 3D). Compared with the BPS group, the levels of T-CHO, TG, LDL-C, ALT, and AST in the PUE + BPS group were significantly decreased, and the level of HDL-C was significantly increased, as shown in Figure 3. These results indicate that long-term exposure to high doses of BPS can lead to lipid accumulation and liver damage and that PUE can alleviate BPS-induced liver damage.

### 3.3. Changes in Oxidative Stress Levels in Liver Tissues of Mice in Each Group

**Figure 4:**
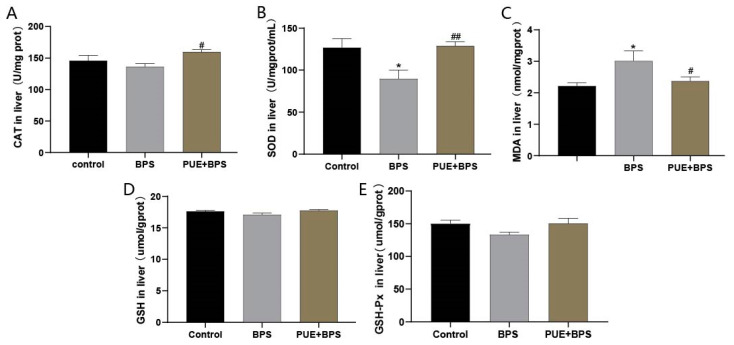
Using liver homogenate to detect liver oxidative stress indicators, (**A**) liver catalase level; (**B**) liver superoxide dismutase level; (**C**) liver malondialdehyde level; (**D**) liver reduced glutathione level; (**E**) liver glutathione levels. “*” represents the BPS group compared with the control group, *p* < 0.05; “#” and “##” represent the BPS group compared with the PUE + BPS group, *p* < 0.05, *p* < 0.01, separately; *n* = 5.

To determine whether BPS enhanced oxidative stress, we used liver homogenate supernatants to detect oxidative stress levels. Compared with the control group, the level of SOD in the BPS group was significantly decreased (Figure 4B), the level of MDA was significantly increased (Figure 4C), and the levels of CAT, GSH, and GSH-peroxidase (Px) were not significantly different from the control group (Figure 4A,D,E). Compared with the BPS group, the levels of CAT and SOD in the PUE + BPS group were significantly increased (Figure 4A,B), and the level of MDA was significantly decreased (Figure 4C). This shows that BPS can induce oxidative stress in the mice liver and that SOD and MDA are more sensitive to BPS. Interventions with PUE can significantly alleviate stress-related injuries.

### 3.4. Changes in H&E and Oil Red O Staining of Liver Tissues in Each Group

**Figure 5:**
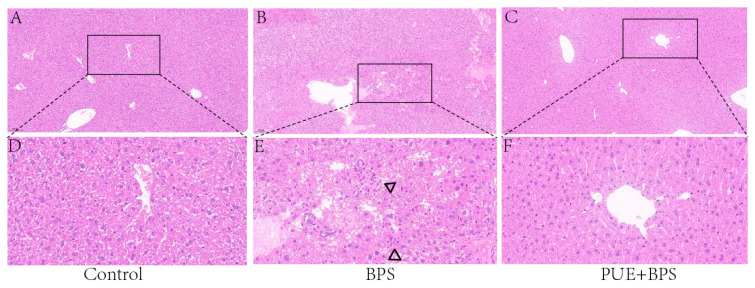
Figure (**A**–**C**) is representative schematic diagram of liver H&E staining, microscope scale 100 μm; (**D**–**F**) is the magnified view in “□”; “△” represents inflammatory cell infiltration.

**Figure 6:**
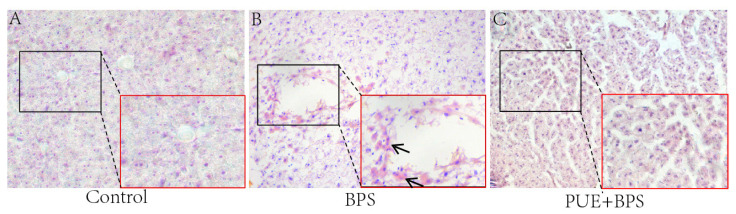
Figure (**A**–**C**) is representative schematic diagram of liver oil red O staining, microscope scale 100 μm; in (**B**), “↑” refers to red fat droplets.

H&E staining showed that the hepatocytes in the control and PUE + BPS group were arranged radially around the central vein, and the nucleus was centered (Figure 5D,F). The liver cells in the BPS group were disordered, the cells were dispersed and uneven in size, and there is an inflammatory cell infiltration (Figure 5E). The liver cells in the PUE + BPS group were arranged in an orderly manner without obvious inflammatory cell infiltration (Figure 5F). Oil Red O staining showed no obvious fat droplets in the control group (Figure 6A); however, a large number of fat droplets were observed in the BPS group (Figure 6B). Compared to the BPS group, no obvious fat droplets were observed in the PUE + BPS group (Figure 6C).

### 3.5. The mRNA Expression of SREBP1C, FASN, Cpt1a, Cpt1b, PPARγ, PPARα, CD36, IL-1β, and TNF-α in the Liver Tissues of Each Group of Mice

**Figure 7:**
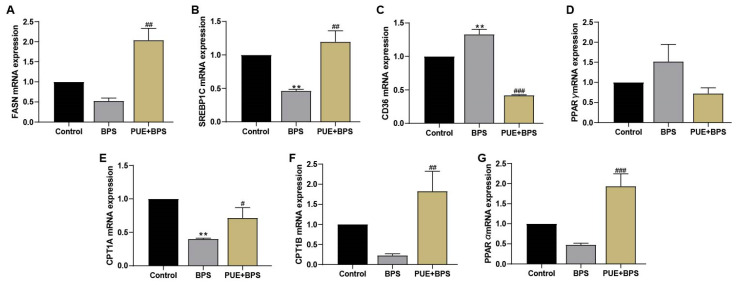
Total RNA was extracted from the liver for qPCR detection of lipid metabolism-related genes. (**A**–**D**) were related genes involved in lipid metabolism synthesis, and (**E**–**G**) were related genes promoting fatty acid metabolism. “**“ represents the BPS group compared with the control group, *p* < 0.01; “#”, “##” and “###” represents the BPS group compared with the PUE + BPS group, *p* < 0.05, *p* < 0.01 and *p* < 0.001, separately; *n* = 3.

**Figure 8:**
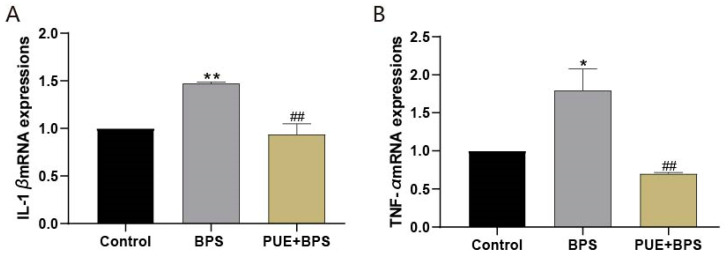
Total RNA was extracted from the liver for qPCR detection of inflammatory factor-related genes. “*” and “**” represents BPS group compared with control group, *p* < 0.05, *p* < 0.01, separately; “##“ represents BPS group compared with PUE + BPS group, *p* < 0.01. *n* = 3.

Compared to the control group, the mRNA expression of FASN and SREBP1C, which are involved in lipid synthesis, decreased in the BPS group, and that of SREBP1C decreased significantly (Figure 7A,B). The expression of CD36 mRNA, which is also involved in lipid synthesis, was significantly increased (Figure 7C), and the expression of PPAR γ mRNA was increased but not statistically significant (*p* > 0.05) (Figure 7D). The mRNA expression of PPARα, Cpt1a, and Cpt1b, which are involved in promoting fatty acid oxidation, decreased (Figure 7E–G), with the decrease in Cpt1a being the most significant (*p* < 0.05). Compared with the BPS group, the expression of PPAR γ in the PUE + BPS group decreased, while the expression of CD36 mRNA decreased significantly, and the expression of PPARα, Cpt1b, SREBP1C, and FASN mRNA increased significantly. The mRNA expression of IL-1β and TNF-α in the BPS group was significantly higher than that in the control group (Figure 8A,B), while the mRNA expression in the PUE + BPS group was significantly decreased. The quantitative PCR (qPCR) results preliminarily indicated that long-term exposure to BPS may induce liver inflammation in mice. In addition, the CD36 and PPAR γ genes involved in lipid synthesis in the liver were stimulated, and the PPAα genes that promote fatty acid oxidation and their target genes Cpt1 a and Cpt1 b were inhibited. The PUE intervention maintained lipid synthesis genes at normal levels and protected FA oxidation genes from inhibition. This suggests that long-term exposure to BPS in mice can cause the inflammatory response liver lipid accumulation by increasing lipid synthesis and reducing FA metabolism, resulting in obesity or lipid metabolism disorders in mice, which PUE can alleviate. 

### 3.6. Changes in Protein Levels of SREBP1C, FASN, Cpt1b, and PPARγ in the Liver Tissues of Mice in Each Group

**Figure 9:**
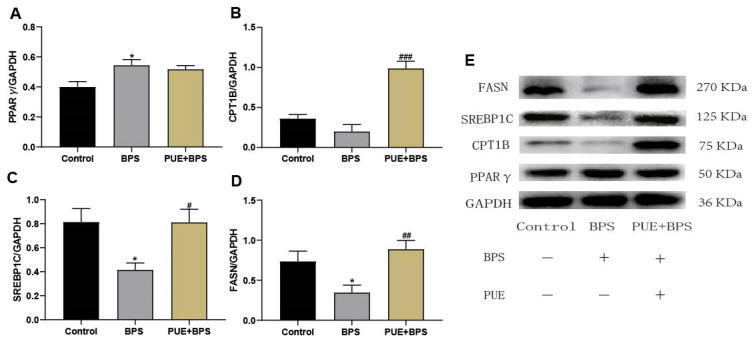
Extract liver protein for Western blot detection, “*” represents the BPS group compared with the control group, *p* < 0.05; “#”, “##” and “###” represents the BPS group compared with the PUE + BPS group, *p* < 0.05, *p* < 0.01, and *p* < 0.001, separately. *n* = 3.

Consistent with the qPCR results, the protein expression of PPARγ in the BPS group significantly increased (Figure 9A), while the protein expression of FASN and SREBP1C significantly decreased (Figure 9C,D). The protein expression of Cpt1b was relatively low (Figure 9B), but the difference was not statistically significant (*p* > 0.05). It shows that BPS causes the disorder of lipid synthesis and may increase lipid synthesis through PPARγ to cause liver lipid accumulation. Compared to the BPS group, the expression of FASN and SREBP1C proteins in the PUE + BPS group significantly increased, which was consistent with the control group level. The protein expression of Cpt1b, which promotes lipid metabolism, was increased in the PUE + BPS group, indicating that PUE could restore normal lipid metabolism in the liver of mice and reduce lipid accumulation by promoting lipid oxidation, confirming the protective effect of PUE. In all events, the results of Western blotting further indicate that BPS caused liver lipid metabolism disorders in mice.

## 4. Discussion

As a substitute for BPA, BPS is widely distributed in everyday environments. With the development of research, more BPS toxicity has been observed. BPS can cause endocrine disruption [30], depression [31], and obesity [32], which can cause different degrees of health hazards to the human body. In a previous study, we confirmed that BPS can induce reproductive toxicity in mice [33] and neurotoxicity in male mice [34]. The liver is a key organ in maintaining local and systemic metabolic homeostasis [35]. Several studies have shown that bisphenols such as BPA, BPF, and BPS can cause lipid accumulation in the liver and obesity [36,37]. In our study, after BPS exposure, the number of inflammatory cells in the liver of mice increased, the expression of IL-1β and TNF-α was upregulated, and the activities of ALT and AST increased, which proved the occurrence of liver inflammation and injury. Histological staining confirmed this finding, which is consistent with the results of perinatal exposure to bisphenols in mice studied by Meng et al. [38]. BPS exposure causes oxidative stress in the liver, increases MDA levels, and reduces SOD levels. Other researchers [39] obtained the same results. The levels of TG, T-CHO, and LDL-C in the BPS group were increased, and the content of HDL-C was decreased, suggesting that the lipid metabolism of mice was altered, and the results of Oil Red O staining verified the increase in lipid droplets in vitro. Inflammation, stress, and injury occurred simultaneously in the livers of mice, and these phenomena were alleviated by PUE treatment. PUE is a dietary isoflavone that can effectively alleviate depressive behavior in mice fed a high-fat diet and downregulate IL-1β levels [40]. PUE can also significantly reduce TNF-α levels in obese mice to reduce obesity-induced inflammation and dyslipidemia [41]. Our results showed that PUE alleviated inflammation and dyslipidemia. After long-term BPS exposure, the liver coefficient of the mice increased, and the weight gain trend was obvious. Interestingly, mRNA and protein levels of FASN and SREBP1C, which are involved in lipid synthesis, were significantly lower in the BPS group. According to the mechanism of lipid synthesis, the levels of TG and T-CHO and the content of lipid droplets in this group of mice should be reduced; however, our results showed that even if the expression of FASN and SREBP1 C decreased, it did not affect TG and T-CHO, and the lipid droplet content was higher than those of the control and PUE groups. As a key factor involved in lipid synthesis, CD36 plays a role in the high-affinity tissue uptake of FAs and promotes lipid accumulation and metabolic dysfunction under excessive fat supply [42]. BPS exposure stimulated a significant increase in CD36 mRNA levels. PPAR γ, which also regulates lipid synthesis, had its protein expression significantly increased, indicating that long-term exposure to BPS could lead to lipid accumulation in male mice, which was not regulated by FASN and SREBPs, but mainly due to the overexpression of CD36 and PPAR γ. Zhuang et al. [43] confirmed that overexpression of CD36 or PPAR γ promotes lipid accumulation induced by oxidized low-density lipoproteins. Our study also showed that the mRNA expression of PPARα, Cpt1a, and Cpt1b, which are involved in lipid metabolism, was decreased in the BPS group, consistent with the decrease in Cpt1b protein expression. After PUE treatment, the mRNA and protein expression levels of SREBP1C and FASN, which are involved in lipid synthesis, were similar to those in the control group. The mRNA or protein levels of PPARα and Cpt1b involved in lipid metabolism were much higher than those in the BPS group, indicating that PUE stimulated the expression of genes and proteins related to lipid metabolism. Che et al. [44] studied the changes in lipid metabolism-related indicators between normal liver and hepatocellular carcinoma samples with FASN inhibition and overexpression and showed that the genes involved in the biosynthesis of TG and FA were upregulated in FASN-inhibited hepatocellular carcinoma samples, but the gene expression data of FASN-inhibited normal liver tissues did not occur. In contrast, the stress response was upregulated. Researchers believe that FASN plays a role in protecting healthy livers from stress. In our experiment, the expression of FASN was inhibited and the expression of SREBP1C was not upregulated in normal mice after exposure to BPS. Disorders of liver lipid metabolism in mice may increase the risk of hepatocellular carcinoma. After PUE intervention, the expression levels of FASN and SREBP1 C returned to those in the control group, which may indicate that PUE protects the liver from damage. The synthesis of TG and T-CHO did not decrease after inhibition of FASN and SREBP1C in the BPS group, which was due to the overexpression of CD36 and PPAR γ and the inhibition of PPARα, Cpt1a, and Cpt1b. B-cell lymphoma 6 (BCL6) protein inhibits CD36 transcription by directly binding to the CD36 promoter region under physiological conditions and inhibits the progression of non-alcoholic fatty liver disease in mice in a CD36-dependent manner, including disordered lipid accumulation and glucose metabolism [45]. Whether PUE alleviates lipid accumulation and metabolism consistently with BCL6 requires further investigation.

## 5. Conclusions

In summary, after BPS exposure, mice developed lipid accumulation and metabolic disorders. In this series of processes, CD36, PPARγ, and PPARα may be the most important factors, which is consistent with the results of previous studies [46]. Regulating the RARα-PPARγ-CD36 cascade can reduce lipid accumulation and lipotoxicity in hepatocytes and reduce nonalcoholic steatohepatitis in mice. In future, we will conduct in vitro studies to explore the specific mechanisms underlying BPS-induced lipid metabolism disorders. The harmful effects of BPS on the human body warrant further studies, and the pharmacological effects of PUE remain to be explored. This experiment provides a reference value for future studies on BPS and PUE.

## Figures and Tables

**Table 1:** The primer sequences of the lipid metabolism genes.

Gene	Primer	Sequences (5′ → 3′)
FASN	Forward	CGGAGTCGCTTGAGTATA
	Reverse	CACAGGGACCGAGTAATG
Cpt1a	Forward	GGAGAATGCCAGGAGGTC
	Reverse	GGTGTCAAATGGGAAGGA
PPARγ	Forward	CACAATGCCATCAGGTTT
	Reverse	CTCGCAGATCAGCAGACT
PPARα	Forward	CAAGTGCCTGTCTGTCGG
	Reverse	CAGGTAGGCTTCGTGGAT
CD36	Forward	ATTCTCATGCCAGTCGGA
	Reverse	TTTGCTGCTGTTCTTTGC
SREBP-1c	Forward	TCTCCTAGAGCGAGCGTT
	Reverse	AGGGCATCTGAGAACTCC
Cpt1b	Forward	AGACTGTGCGTTCCTGTA
	Reverse	TTGGAGACGATGTAAAGG
GAPDH	Forward	CCTCGTCCCGTAGACAAAATG
	Reverse	TGAGGTCAATGAAGGGGTCGT
IL-1β	Forward	GATGGCTTATTACAGTGGC
	Reverse	TAGTGGTGGTCGGAGATT
TNF-α	Forward	ACGCTCTTCTGCCTGCTG
	Reverse	CTTGTCACTCGGGGTTCG
β-actin	Forward	CACCCGCGAGTACAACCTC
	Reverse	CCCATACCCACCATCACACC

## Data Availability

Data are available from the corresponding author by request.

## References

[1] Wu L.H., Zhang X.M., Wang F., Gao C.J., Chen D., Palumbo J.R., Guo Y., Zeng E.Y. (2018). Occurrence of bisphenol S in the environment and implications for human exposure: A short review. Sci. Total Environ..

[2] Glausiusz J. (2014). Toxicology: The plastics puzzle. Nature.

[3] Wang H., Gao R., Liang W., Wei S., Zhou Y., Zeng F. (2022). Assessment of BPA and BPS exposure in the general population in Guangzhou, China—Estimation of daily intakes based on urinary metabolites. Environ. Pollut..

[4] Ndaw S., Remy A., Denis F., Marsan P., Jargot D., Robert A. (2018). Occupational exposure of cashiers to bisphenol S via thermal paper. Toxicol. Lett..

[5] Profita M., Fabbri E., Spisni E., Valbonesi P. (2021). Comparing effects and action mechanisms of BPA and BPS on HTR-8/SVneo placental cellsdagger. Biol. Reprod..

[6] Jia L.L., Luan Y.L., Shen H.M., Guo Y. (2022). Long-term stability of several endocrine disruptors in the first morning urine samples and their associations with lifestyle characteristics. Sci. Total Environ..

[7] Meng Z., Wang D., Liu W., Li R., Yan S., Jia M., Zhang L., Zhou Z., Zhu W. (2019). Perinatal exposure to Bisphenol S (BPS) promotes obesity development by interfering with lipid and glucose metabolism in male mouse offspring. Environ. Res..

[8] Hong X., Zhou Y., Zhu Z., Li Y., Li Z., Zhang Y., Hu X., Zhu F., Wang Y., Fang M. (2023). Environmental endocrine disruptor Bisphenol A induces metabolic derailment and obesity via upregulating IL-17A in adipocytes. Environ. Int..

[9] Zhang Y., Dong T., Hu W., Wang X., Xu B., Lin Z., Hofer T., Stefanoff P., Chen Y., Wang X. (2019). Association between exposure to a mixture of phenols, pesticides, and phthalates and obesity: Comparison of three statistical models. Environ. Int..

[10] Meng Z., Wang D., Yan S., Li R., Yan J., Teng M., Zhou Z., Zhu W. (2018). Effects of perinatal exposure to BPA and its alternatives (BPS, BPF and BPAF) on hepatic lipid and glucose homeostasis in female mice adolescent offspring. Chemosphere.

[11] Reina-Pérez I., Olivas-Martínez A., Mustieles V., Ruiz-Ojeda F.J., Molina-Molina J.M., Olea N., Fernández M.F. (2021). Bisphenol F and bisphenol S promote lipid accumulation and adipogenesis in human adipose-derived stem cells. Food Chem. Toxicol..

[12] Gianfrancesco M.A., Paquot N., Piette J., Legrand-Poels S. (2018). Lipid bilayer stress in obesity-linked inflammatory and metabolic disorders. Biochem. Pharmacol..

[13] Nagarajan S.R., Cross E., Sanna F., Hodson L. (2022). Dysregulation of hepatic metabolism with obesity: Factors influencing glucose and lipid metabolism. Proc. Nutr. Soc..

[14] Trefts E., Gannon M., Wasserman D.H. (2017). The liver. Curr. Biol..

[15] Horton J.D., Goldstein J.L., Brown M.S. (2002). SREBPs: Activators of the complete program of cholesterol and fatty acid synthesis in the liver. J. Clin. Investig..

[16] Zheng Q.W., Ding X.F., Cao H.J., Ni Q.Z., Zhu B., Ma N., Zhang F.K., Wang Y.K., Xu S., Chen T.W. (2021). Ochratoxin A Induces Steatosis via PPARgamma-CD36 Axis. Toxins.

[17] Zeng H., Qin H., Liao M., Zheng E., Luo X., Xiao A., Li Y., Chen L., Wei L., Zhao L. (2022). CD36 promotes de novo lipogenesis in hepatocytes through INSIG2-dependent SREBP1 processing. Mol. Metab..

[18] Bougarne N., Weyers B., Desmet S.J., Deckers J., Ray D.W., Staels B., De Bosscher K. (2018). Molecular Actions of PPARalpha in Lipid Metabolism and Inflammation. Endocr. Rev..

[19] Naiman S., Huynh F.K., Gil R., Glick Y., Shahar Y., Touitou N., Nahum L., Avivi M.Y., Roichman A., Kanfi Y. (2019). SIRT6 Promotes Hepatic Beta-Oxidation via Activation of PPARalpha. Cell Rep..

[20] Xiong S., Wang W., Kenzior A., Olsen L., Krishnan J., Persons J., Medley K., Peuß R., Wang Y., Chen S. (2022). Enhanced lipogenesis through Ppargamma helps cavefish adapt to food scarcity. Curr. Biol..

[21] Li Z., Liu T., Feng Y., Tong Y., Jia Y., Wang C., Cui R., Qu K., Liu C., Zhang J. (2022). PPARgamma Alleviates Sepsis-Induced Liver Injury by Inhibiting Hepatocyte Pyroptosis via Inhibition of the ROS/TXNIP/NLRP3 Signaling Pathway. Oxid. Med. Cell Longev..

[22] Park M., Yoo J.H., Lee Y.S., Lee H.J. (2019). Lonicera caerulea Extract Attenuates Non-Alcoholic Fatty Liver Disease in Free Fatty Acid-Induced HepG2 Hepatocytes and in High Fat Diet-Fed Mice. Nutrients.

[23] Zhou Y.X., Zhang H., Peng C. (2014). Puerarin: A review of pharmacological effects. Phytother. Res..

[24] Anukulthanakorn K., Parhar I.S., Jaroenporn S., Kitahashi T., Watanbe G., Malaivijitnond S. (2016). Neurotherapeutic Effects of Pueraria mirifica Extract in Early- and Late-Stage Cognitive Impaired Rats. Phytother. Res..

[25] Sun L., Jia H., Yu M., Yang Y., Li J., Tian D., Zhang H., Zou Z. (2021). Salvia miltiorrhiza and Pueraria lobata, two eminent herbs in Xin-Ke-Shu, ameliorate myocardial ischemia partially by modulating the accumulation of free fatty acids in rats. Phytomedicine.

[26] Liu Y.S., Yuan M.H., Zhang C.Y., Liu H.M., Liu J.R., Wei A.L., Ye Q., Zeng B., Li M.F., Guo Y.P. (2021). Puerariae Lobatae radix flavonoids and puerarin alleviate alcoholic liver injury in zebrafish by regulating alcohol and lipid metabolism. Biomed. Pharmacother..

[27] Li Q., Liu W., Zhang H., Chen C., Liu R., Hou H., Luo Q., Yu Q., Ouyang H., Feng Y. (2023). alpha-D-1,3-glucan from Radix Puerariae thomsonii improves NAFLD by regulating the intestinal flora and metabolites. Carbohydr. Polym..

[28] Li Q., Liu W., Feng Y., Hou H., Zhang Z., Yu Q., Zhou Y., Luo Q., Luo Y., Ouyang H. (2022). Radix Puerariae thomsonii polysaccharide (RPP) improves inflammation and lipid peroxidation in alcohol and high-fat diet mice by regulating gut microbiota. Int. J. Biol. Macromol..

[29] Livak K.J., Schmittgen T.D. (2001). Analysis of relative gene expression data using real-time quantitative PCR and the 2^−ΔΔCT^ Method. Methods.

[30] en Braver-Sewradj S.P., van Spronsen R., Hessel EV S. (2020). Substitution of bisphenol A: A review of the carcinogenicity, reproductive toxicity, and endocrine disruption potential of alternative substances. Crit. Rev. Toxicol..

[31] Hao K., Luo J., Sun J., Ge H., Wang Z. (2021). Associations of urinary bisphenol A and its alternatives bisphenol S and F concentrations with depressive symptoms among adults. Chemosphere.

[32] Choi J.Y., Lee J., Huh D.A., Moon K.W. (2022). Urinary bisphenol concentrations and its association with metabolic disorders in the US and Korean populations. Environ. Pollut..

[33] Dai W., He Q.Z., Zhu B.Q., Zeng H.C. (2021). Oxidative stress-mediated apoptosis is involved in bisphenol S-induced reproductive toxicity in male C57BL/6 mice. J. Appl. Toxicol..

[34] Li Y.Z., Wu Z.Y., Zhu B.Q., Wang Y.X., Kan Y.Q., Zeng H.C. (2022). The BDNF-TrkB-CREB Signalling Pathway Is Involved in Bisphenol S-Induced Neurotoxicity in Male Mice by Regulating Methylation. Toxics.

[35] Cheng M.L., Nakib D., Perciani C.T., MacParland S.A. (2021). The immune niche of the liver. Clin. Sci..

[36] Ramskov Tetzlaff C.N., Svingen T., Vinggaard A.M., Rosenmai A.K., Taxvig C. (2020). Bisphenols B, E, F, and S and 4-cumylphenol induce lipid accumulation in mouse adipocytes similarly to bisphenol A. Environ. Toxicol..

[37] Ahn Y.A., Baek H., Choi M., Park J., Son S.J., Seo H.J., Jung J., Seong J.K., Lee J., Kim S. (2020). Adipogenic effects of prenatal exposure to bisphenol S (BPS) in adult F1 male mice. Sci. Total Environ..

[38] Meng Z., Tian S., Yan J., Jia M., Yan S., Li R., Zhang R., Zhu W., Zhou Z. (2019). Effects of perinatal exposure to BPA, BPF and BPAF on liver function in male mouse offspring involving in oxidative damage and metabolic disorder. Environ. Pollut..

[39] Wang C., He J., Xu T., Han H., Zhu Z., Meng L., Pang Q., Fan R. (2021). Bisphenol A(BPA), BPS and BPB-induced oxidative stress and apoptosis mediated by mitochondria in human neuroblastoma cell lines. Ecotoxicol. Environ. Saf..

[40] Liu Y., Hu Z., Wang J., Liao Y., Shu L. (2023). Puerarin alleviates depressive-like behaviors in high-fat diet-induced diabetic mice via modulating hippocampal GLP-1R/BDNF/TrkB signaling. Nutr. Neurosci..

[41] Noh J.W., Yang H.K., Jun M.S., Lee B.C. (2022). Puerarin Attenuates Obesity-Induced Inflammation and Dyslipidemia by Regulating Macrophages and TNF-Alpha in Obese Mice. Biomedicines.

[42] Pepino M.Y., Kuda O., Samovski D., Abumrad N.A. (2014). Structure-function of CD36 and importance of fatty acid signal transduction in fat metabolism. Annu. Rev. Nutr..

[43] Zhuang J.L., Liu Y.Y., Li Z.Z., Zhuang Q.Z., Tang W.Z., Xiong Y., Huang X.Z. (2021). Amentoflavone prevents ox-LDL-induced lipid accumulation by suppressing the PPARgamma/CD36 signal pathway. Toxicol. Appl. Pharmacol..

[44] Che L., Chi W., Qiao Y., Zhang J., Song X., Liu Y., Li L., Jia J., Pilo M.G., Wang J. (2020). Cholesterol biosynthesis supports the growth of hepatocarcinoma lesions depleted of fatty acid synthase in mice and humans. Gut.

[45] Zhang H., Li Y., Zhang C., Huang K., Zhao J., Le S., Jiang L., Liu H., Yang P., Xiao X. (2022). B-cell lymphoma 6 alleviates nonalcoholic fatty liver disease in mice through suppression of fatty acid transporter CD36. Cell Death Dis..

[46] Zhao Z., Deng Z.T., Huang S., Ning M., Feng Y., Shen Y., Zhao Q.S., Leng Y. (2022). Alisol B Alleviates Hepatocyte Lipid Accumulation and Lipotoxicity via Regulating RARalpha-PPARgamma-CD36 Cascade and Attenuates Non-Alcoholic Steatohepatitis in Mice. Nutrients.

